# Psychometric adequacy of the Persian adapted version of the Tilburg pregnancy distress scale (P-TPDS)

**DOI:** 10.1186/s12884-021-03745-1

**Published:** 2021-04-09

**Authors:** Leili Salehi, Zoherh Mohmoodi, Fatemeh Rajati, Victor Pop

**Affiliations:** 1 Research Center for Health, Safety and Environment, Department of Health Education and Promotion, Alborz University of Medical Sciences, Karaj, Iran; 2 Research Center for Social Determinants of Health, Alborz University of Medical Sciences, Karaj, Iran; 3 Department of Health Education and Promotion, School of Health, Research Center for Environmental Determinants of Health, Kermanshah University of Medical Sciences, Kermanshah, Iran; 4 Department of Medical and Clinical Psychology, Tilburg University, Tilburg, Netherlands

**Keywords:** Pregnancy distress, Pregnancy distress scale, Psychometric

## Abstract

**Background:**

Pregnancy distress is a combination of anxiety, stress, and depression during pregnancy. The first step in preventing pregnancy distress is to identify women at risk. The present study assessed adaptation and psychometric adequency of the Persian Adapted Version of Tilburg Pregnancy Distress Scale (P-TPDS).

**Methods:**

By Brislin’s translation guidelines, TPDS was translated to Persian. This was followed by determining the face validity of P-TPDS and evaluating construct validity using exploratory and confirmatory factor analyses. The Cronbach’s alpha coefficients and intra-class correlation coefficient (ICC) were used to estimate reliability.

**Results:**

A final 16-item scale was loaded on four distinct constructs jointly accounting for 59.62% of variance. The factors were labelled as delivery-related worries, partner involvement, pregnancy-related worries, and social-related worries. The alpha coefficients for P-TPDS subscales ranged from 0.85 to 0.91 and ICC ranged from 0.70 to 0.77. All comparative indices of the model including CFI, IFI, NFI, and NNFI were above 0.9 showing the goodness of fit for the data with a RMSEA of 0.04, lower bound: 0.038.

**Conclusions:**

The Persian adapted version of TPDS (P-TPDS) is a reliable and valid scale for assessing pregnancy distress among pregnant women in Iran.

**Supplementary Information:**

The online version contains supplementary material available at 10.1186/s12884-021-03745-1.

## Background

Mental health is among main public health issues, which not only have been neglected as compared to physical health issues but have also been neglected in both budget allocation and access to services. Pregnancy is an intricate process of physical and physiological changes causing, in some people, psychological distress [1]. Perinatal-specific maternal distress has been described as “*a woman’s response to transition to motherhood, which includes changes to their bodies, roles, relationships, and social circumstances, [and her response to] birth experiences as well as the demands, challenges, losses and gains associated with being a new mother*” [2]. Specifically, pregnant women can experience distress with regard to fears and worries related to delivery and childbirth [3], bodily changes, and the health of their baby [4]. This type of distress occurs on a continuum of severity, ranging from minor to severe levels of worries and stress [2, 5].

The estimated prevalence of depressive symptoms is 7–19% during pregnancy [6–9] and 18–25% for symptoms of anxiety [10]; and such symptoms can have negative impacts on obstetric outcome, fetal development, and neurodevelopment of the offspring [11]. The first step in preventing pregnancy distress, therefore, is to identify women at risk.

Several instruments, such as Beck Anxiety Inventory, Edinburg Postpartum Depression Scale, and Prenatal Perceived Stress Questionnaire, have been developed to assess mental health status in pregnant women; however, they are unidimensional or nonspecific for pregnancy. Moreover, having been developed by clinicians/researchers, they are not based on the experiences of the target group, i.e., pregnant women. The golden standard for developing questionnaires is to start from focus group interviews. Therefore, we need multidimensional tools specific for pregnant women to measure pregnancy distress [12]. A great deal of attention has been paid to the use of prenatal instruments as valid and sensitive tools for assessing prenatal mental health [13]. One of such tools, which assesses overall stress, is the Cambridge Worry Scale, addressing general concerns not stressful life events. In fact, it focuses on anxiety rather than stress [14]. The Prenatal Distress Questionnaire (PDQ), designed by Yalis and Lobel in 1999, focuses on prenatal stressors by addressing maternal anxiety and fears during the weeks 22–28 of pregnancy. The said questionnaire measures maternal stress concerning clinical interventions in pregnancy, which can lead to preterm labor or postpartum complications; thus, postpartum potential stressors are neglected [15]. One of the most frequently used instruments in the world is the Pregnancy-Related Anxiety Questionnaire (PRAQ), developed by Van den Bergh [16] and revised by Huizink and colleagues [17] into a feasible abbreviated 10-item version (PRAQ-10) with three subscales, namely, “fear of labor”, “worries about child health”, and “concerns about maternal changes appearance”. The questionnaire measures mother’s anxiety related to pregnancy, delivery, and child-related consequences. However, the construct of this questionnaire is not based on focus group interviews either, since it was originally developed only for primiparous women and did not contain a “partner involvement” dimension.

The Tilburg Pregnancy Distress Scale (TPDS)(Supplementary file no.1) is a comprehensive scale that focuses on distress among pregnant women. It was developed based on in-depth qualitative studies including (primi- and multiparous) pregnant women, women who recently gave birth, midwives, obstetricians, and maternity nurses [4]. The TPDS is a 16-item instrument, designed and validated by Pop et al. in 2011 in the Netherlands and then translated and validated in several countries [11, 13, 18]. Instrument efficacy has been evaluated in each trimester of pregnancy [19]. However, thus far, psychometric adequacy of a P-TPDS have not been assessed in Persian culture. Therefore, the current study aimed to assess psychometric adequacy of an P-TPDS version.

## Methods

### Design

This methodological study was performed with the participation of pregnant women referring to Karaj health care centers during four phases.

### Phase 1

#### Translation

The original version of the TPDS was translated into Persian according to modified Brislin’s translation model [20]. In line with this, initially, the English version of TPDS was translated into Persian by two competent English-Persian translators independently. Then, a third competent translator was assigned to preparing a final Persian translation by merging the two original Persian translations. This was followed by getting two other translators being native-like in English to back-translate the final Persian translation into English. Finally, the English back-translations were compared to the original English version and the discrepancies between the former and the latter in terms of accuracy of messages were attended to, which entailed that the final Persian translation be modified in some instances.

### Phase2

#### Content and face validity

Content validity was assessed qualitatively by asking ten experts (in such fields as clinical psychology, health psychology, gynecology and health reproduction) for their opinions; and face validity was assessed using the opinions of 20 pregnant women.

### Phase 3

During the third phase, the scale was sent out for completion to pregnant women, who helped assess construct validity and reliability.

#### Participants

The study population included 468 people (225 for explanatory factor analysis and 243 for confirmatory factor analysis) from six health care centers. The rule of thumb, such as 5 or 10 participants per item, was applied for calculating the sample size [21]. In order to select the sample, at first, a list of the health care centers located in Karaj was prepared. Then, six centers were picked up at random; and in each center, the convenience sampling method was used to select the subjects meeting the inclusion criteria.

#### Inclusion criteria

Inclusion criteria were age over 18 years, spontaneous pregnancy without induction, absence of chronic disease with no history of infertility, and voluntary participation. The questionnaires were completed by women between 6- and 35-weeks’ gestation.

The first author (LS) administered the survey questionnaire, and she was available to answer possible questions. Exploratory and confirmatory factor analyses were used to assess the construct validity of this scale.

This study was approved by the Ethics Committee of Alborz University of Medical Sciences. (Ethical Code: IR.Abzums.Rec.1398.215).

### Construct validity

Exploratory and confirmatory factor analyses were used to assess the construct validity of this scale.

#### Exploratory factor analysis (EFA)

The principal component analysis with varimax rotation was used for exploratory factor analysis. Also, Kiser–Meyer-Olkin (KMO) and Bartlett’s Tests of Sphericity were used to assess the adequacy of the sample for the factor analysis. Consequently, factor loadings equal to or above 0.3 were considered appropriate [22].

#### Confirmatory factor analysis (CFA)

CFA was used to assess the fitness of the model. χ2/df, comparative fit index (CFI), incremental fit index (IFI), normed fit index (NFI), non-normed fit index (NNFI), root mean square error of approximation (RMSEA), and standardized root mean square residual (SRMR) were used as fitness indices.

### Reliability

For reliability, test-retest reliability and Cronbach’s alpha coefficient were used; the minimum sample size for test-retest reliability is recommended to be 15 subjects [23]. Thus, using random sampling, we selected 30 subjects to fill in the same questionnaires two times (2 weeks’ interval). To determine the reliability of the questionnaire, the Cronbach’s alpha and intra-class correlation coefficient (ICC) were calculated for each factor. ICC of 0.6 or above was considered acceptable [24]; and the acceptable level of Cronbach’s alpha coefficient was set at 0.7 [25].

#### Concurrent validity

During this phase, the concurrent validity of the scale was evaluated. Concurrent validity measures how well a new test compares to a well-established test [26]. Concurrent validity of the TPDS was tested by correlating (Pearson correlations, two-tailed) the TPDS with the EPDS and the PRAQ-10.

#### Edinburgh’s postnatal depression scale (EPDS)

The most commonly used and validated questionnaire to identify women at risk for perinatal depression is the 10-item Edinburgh (postnatal) Depression Scale (EDS) [27]. The EDS has been validated in pregnant women, showing adequate sensitivity and specificity to detect women at risk for depression during pregnancy [28]. In their review, O’Connor et al. (2016) concluded that the EDS is a frequently used and widely applicable instrument to screen for perinatal depression [29].

The EPDS consists of 10 questions scored on a 4-point Likert scale, ranging from 0 to 3 (in terms of symptom severity). The scores range from 0 to 30, where scores of 0–9 indicate no depression, scores of 10–12 show the risk for depression, and scores of 13 and above indicate depression [30]. This scale has been validated in Iran among pregnant women [31].

#### Pregnancy-related anxiety questionnaire (PRAQ-10)

This questionnaire has been revised by Huizink et al. for assessing anxiety during pregnancy. It includes 10 items and 3 structural factors, i.e., fear of delivery with 3 items (1, 2, 6), anxiety about giving birth to a physically or mentally disabled child with 4 items (4, 9, 10, 11), and anxiety about physical changes with 3 items (3, 5, 7). Each item is scored based on a 5-point Likert scale. The total score of the questionnaire is the sum of each item’s score with no defined cut-off point [17]. This scale has been validated in Iran among pregnant women [32].

### Outcome measurements

#### Tilburg pregnancy distress scale (TDPS)

TDPS comprises 16 items and two subscales. The items of the questionnaire are scored using weighted sum scores (multiplying the score of each item into its factor loading and then summing all of them).

Negative affect subscale (NA): This subscale consists of 11 items: 3, 5, 6, 7, 9, 10, 11, 12, 13, 14, and 16. The lowest score obtained from this subscale is 0 and the highest score is 33.Partner involvement subscale (PI): This subscale consists of 5 items: 1, 2, 4, 8, and 15. The scores of this subscale range from 0 to 15. According to this scale, each item is graded based on a four-point Likert scale from 0 (very often) to 3 (rarely or never). Items 3, 5, 6, 7, 9, 10, 11, 12, 13, 14, and 16 in the scale are reverse graded. The total scale scores from 0 to 48. The cut-off points are calculated according to the 90th percentile of the total scores of the scale and subscales. The scores above the cut-off points indicate those pregnant women who are at risk regarding distress. The Cronbach’s alpha coefficient of the total scale (original scale) is 0.78, and each subscale is 0.80 [4].

### Data analysis

The Statistical Package for Social Sciences, version 21.0 (SPSS IBM Corp), was used for explanatory factor analysis and other statistical analyses except CFA. CFA was performed using the LISREL 8.80 for Windows.

## Results

### Demographic characteristics

Out of all 468 pregnant women who participated in the study, 225 were studied for EFA, and 243 for CFA. The mean age of participants was 30.78 ± 4.68 in the EFA group and 28.76 in the CFA group. At first, all the prenatal care centers in Karaj were identified to select the samples and then 6 prenatal care centers were selected randomly (simple random sampling).

Characteristics of the study samples are listed in the Table 1.

**Table 1:** Demographic characteristics of the study participant (*n* = 468)

	Sample of EFA(***n*** = 225)		Sample of CFA (***n*** = 243)	
	Mean (SD)	N (%)	Mean (SD)	N (%)
**Age (y)**	30.78 ± 4.68		28.76 ± 6.26	
**Spouse age (y)**	36.21 ± 3.25		36.01 ± 2.22	
**Gestational age(w)**	33.30 ± 1.31		27.01 ± 7.06	
**Education**				
Low		5 (2.22)		12 (4.94)
Medium (high school)		209 (92.89)		213 (87.65)
High		11 (4.89)		18 (7.41)
**Gravida**				
Nulliparous		150 (66.6)		133 (54.5)
Parous		75 (33.3)		110 (45.5)
**Wanted Pregnancy**				
Yes		220 (97.78)		199 (82)
No		5 (2.22)		44 (18)
**Socioeconomic Status**				
High		70 (31.11)		80 (32.92)
Medium		155 (68.89)		155 (63.79)
Low		0		8 (3.29)

### EFA

The KMO was 0.801, and Bartlett’s test of sphericity was significant (1267.651, *P* < 0.001), showing sampling adequacy. The initial analysis indicated a four-factor structure for the instrument. A final 16-item scale was loaded on four distinct constructs that jointly accounted for 59.62 of the variance observed. The factors were labeled as delivery-related worries, partner involvement, pregnancy-related worries, and social-related worries (Table 2).

**Table 2:** Factor loadings based on a principal component analysis extraction with Varimax rotation

	Factor1	Factor 2	Factor 3	Factor 4
Q12. The delivery is troubling me	0.836			
Q11. I often think about choices concerning the delivery	0.763			
Q13. I get very tense hearing stories about deliveries	0.751			
Q5: I worry about the delivery	0.632			
Q10: I am afraid I will lose self-control during delivery	0.573			
Q2: I feel like my partner and I enjoying my pregnancy together		0.976		
Q4: The pregnancy has brought my partner and I close together		0.761		
Q8: I feel supported by my partner		0.757		
Q15: I can really share my feelings with my partner		0.715		
Q1: I’m enjoying my pregnancy			0.625	
Q3: I worry about pregnancy			0.722	
Q6: I worry about health of my baby			0.546	
Q14: I am concerned that the physical discomforts of pregnancy might persist after the childbirth			0.481	
Q16: I worry about gaining too much weight			0.432	
Q7: I worry about my job once the baby is born				0.799
Q9: I worry about our financial situation after child birth				0.719
% of Variance	27.94	17.14	7.97	6.030
Cumulative %	27.94	45.35	53.32	59.63

Factor1: delivery-related worries, Factor2: Partner involvement; Factor3: pregnancy-related worries.; Factor4: worries related social condition

### CFA

**Fig. 1:**
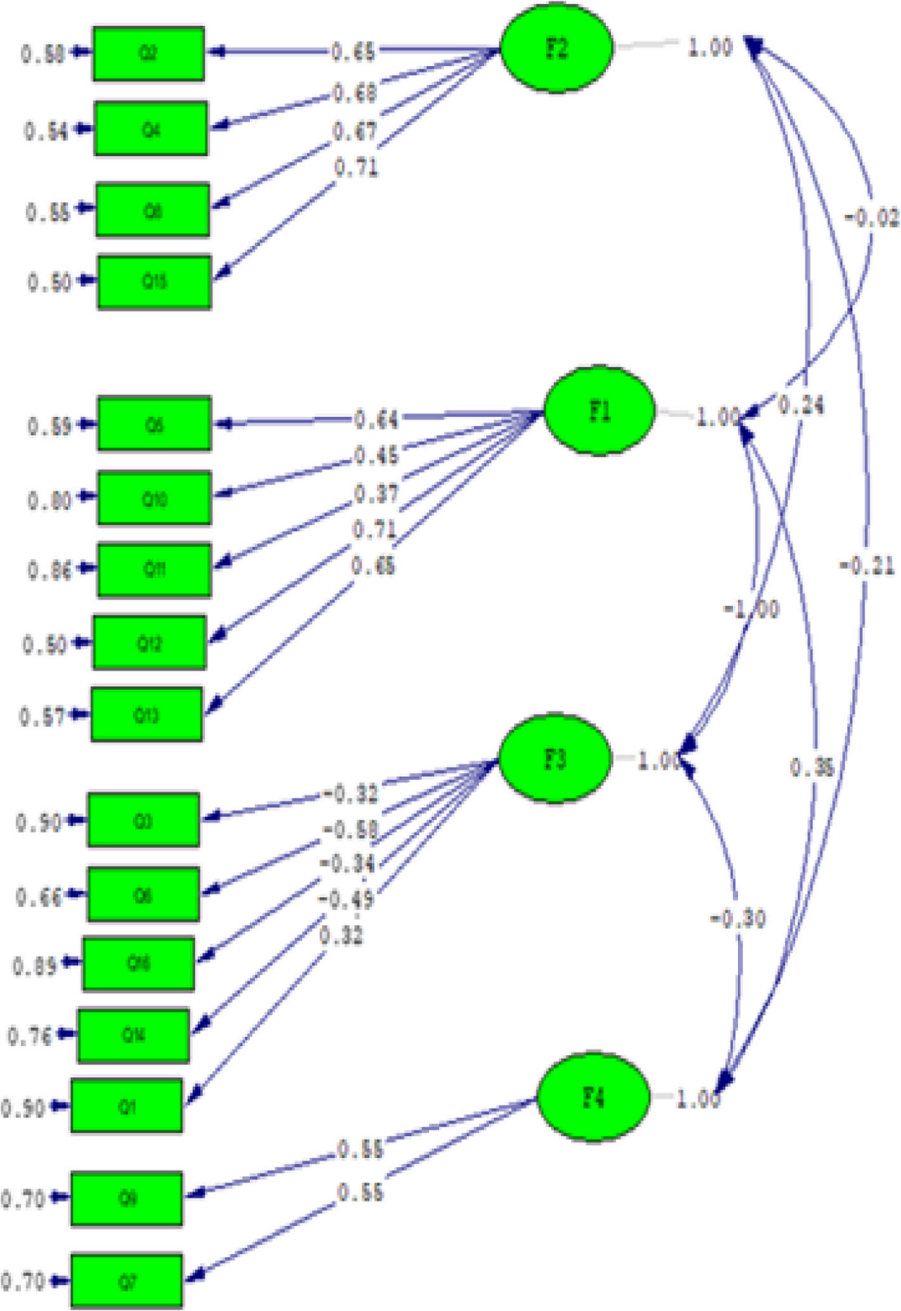
The final confirmatory factor analysis (CFA) model of the P-TPDS

The 16-items scale was used for the CFA to test the model fit. All comparative indices of the model, including CFI, IFI, NFI, and NNFI, were more than 0.9 (0.91, 0.92, 0. 95, and 0.94, respectively), showing the goodness of fit for the data [33]. The RMSEA of the model was 0.04 with a lower bound of 0.038. The SRMR was less than 0.08, confirming an adequate fit for the model [34]. The final model is shown in Fig. 1.

### Concurrent and test re-test assessments

A significant correlation was found between the score of the P-TPDS and the PRAQ (*r* = 0.4900, *P* < 0.001, medium to large effect size) and also the EPDS (*r* = 0.271, *P* < 0.001, low effect size). The test re-test correlation of the P-TPDS in 30 women was 0.82.

### Reliability assessment

The Cronbach’s alpha for four factors ranged from 0.85 to 0.91 and the stability of the scale as assessed by intra-class coefficient (ICC) varied from 0.7 to 0.77. The results are shown in Table 3.

**Table 3:** Cronbach’s alpha and ICC of the P-TDS

Factors	Cronbach’s Alpha Coefficient	ICC
Factor1	0.87	0.70
Factor2	0.91	0.75
Factor3	0.88	0.77
Factor4	0.85	0.72

## Discussion

The current study showed adequate psychometric properties of a Persian adapted version of the TPDS (Supplementary file no.2). We used the direct oblimin rotation method for EFA as the previous studies [4, 12] and obtained four factors based on eigenvalue ≥1 and scree plot. However, one factor lacked items with a factor loading above 0.3; therefore, we repeated the analysis with varimax rotation and extracted four factors.

The first factor included five items, the second one included four items, the third one included five items, and the fourth factor included two items. The factors were termed as delivery-related worries, partner involvement, pregnancy-related worries, and social-related worries.

Our CFA results showed that the four-factor model fit the data reasonably well, χ2(98) =322.71, CFI = 0.89, GFI = 0.89, WFI = 0.88, and RMSEA = 0.05. In line with the current research results, Boekhorst et al. also extracted worries related to pregnancy and worries related to delivery during TPDS psychometric process [19].

Furthermore, Volpato et al., during the process of cross-cultural adaptation and validation of TPDS, identified 3 factors: (1) affection and involvement of the partner, (2) feelings about childbirth, and (3) feelings about the future [18].

It seems that the social conditions of each society during the instrumental psychometric process have caused a small variety of results, so that in Iran, the existence of sanctions, unemployment, and price rise have increased mothers’ worries about their future careers and financial conditions. The two items together in the fourth factor explained 6.304% of the total variance.

According to the findings of the present study, the first factor called delivery-related worries based on its items, with a 27.93% variation, was the most important in the Persian adapted version. Among factors’ items, item 12 (the delivery is troubling me) was the main loading factor. According to the literature, Iranian women are very afraid of labor pains, being the most determining factor in Iranian women’s tendency to give birth by cesarean section [35], so that cesarean section statistics have tripled in the last three decades [36]. In this study, a significant correlation with medium to large effect size was found between the Persian adapted version and PRAQ (*r* = 0.49 referring to 25% explained variance), suggesting that the Persian adapted version does measure pregnancy worries. The correlation between the Persian adapted version and EPDS was also significant (*r* = .27 referring to less than 9% explained variance) with low effect size suggesting that these instruments assess different concepts of mental distress. These findings were in line with other studies [4].

In the current study, the Cronbach’s alpha coefficient indicates good internal consistency. The reliability index ranges 0–1. According to Pallant, the index alpha of 0.7 or above is good for instruments with ten or more items [37]. Unlike other studies, we do not calculate the total Cronbach’s’ alpha value, and it is not necessarily related to the instruments contained in various parts [38].

Given that all study subjects were members of urban government health care centers,the results of the study might not be generalized to all Iranian’s pregnant women.

## Conclusion

The results suggest that the scale has four theoretically meaningful subscales, including delivery-related worries, pregnancy-related worries, partner involvement, and social-related worries. They could be considered as outcome measures in studies aiming to identify effective interventions in order to reduce distress during pregnancy or explore causal relationships between distress during pregnancy and pregnancy outcomes.

## Supplementary Information

**Additional file 1.**

**Additional file 2.**

## Data Availability

The data that support the findings of this study are available from Alborz University of Medical Sciences but restrictions apply to the availability of these data, which were used under license for the current study, and so are not publicly available. Data are however available from the authors upon reasonable request and with permission of Alborz University of Medical Sciences.

## Abbreviations

TPDS: Tilburg pregnancy distress scale
ICC: Intra-class correlation coefficient
PDQ: The prenatal distress questionnaire
KMO: Kiser meyer olkin
CFI: Comparative fit index
IFI: Incremental fit index
NFI: Normed fit index
NNFI: Non-normed fit index
RMSEA: Root mean square error of approximation
SRMR: Standardized root mean square residual
NA: Negative affect
PRAQ: Pregnancy-related anxiety questionnaire
EPDS: Edinburgh postnatal depression scale
ICC: Interclass correlation coefficient
