# Supplementary material for: Psychometric adequacy of the Persian adapted version of the Tilburg pregnancy distress scale (P-TPDS)
Source: BMC Pregnancy Childbirth. 2021 Apr 9;21:281. doi: 10.1186/s12884-021-03745-1 (PMC8033711; doi:10.1186/s12884-021-03745-1)
Supplement: Supplementary file 2 — Additional file 2. [file 12884_2021_3745_MOESM2_ESM.docx]

پرسشنامه دیسترس بارداری تیلبرگ

خانم باردار عزیز پرسشنامه زیر ادراکات شما از بارداری را مورد ارزیابی قرار می‌دهد. لطفا" احساسات خود را در طی 7 روز گذشته در نظر گرفته و مناسب‌ترین گزینه را انتخاب نمایید.

|  | بیشتر اوقات | اغلب اوقات | گاهی اوقات | بندرت یا هیچ‌وقت |
| --- | --- | --- | --- | --- |
| 1.من از بارداریم لذت می‌برم. |  |  |  |  |
| 2.احساسم می‌کنم که من و همسرم هردو از بارداری‌ام لذت می‌بریم |  |  |  |  |
| 3.من در مورد بارداری نگرانم. |  |  |  |  |
| 4.بارداری‌ام باعث نزدیکی بیشتر من و همسرم به هم شده است . |  |  |  |  |
| 5.من در مورد زایمان نگرانم. |  |  |  |  |
| 6.من در مورد سلامتی فرزندم نگرانم. |  |  |  |  |
| 7.من با به دنیا آمدن فرزندم نگران شغلم هستم. |  |  |  |  |
| 8.من از سوی همسرم حمایت می شوم . |  |  |  |  |
| 9.من نگران وضعیت مالیم با به دنیا آمدن فرزندم هستم. |  |  |  |  |
| 10.من می ترسم که در طی زایمان نتوانم خودم را کنترل کنم |  |  |  |  |
| 11.من اغلب در مورد انتخاب نوع زایمان خود فکر می‌کنم. |  |  |  |  |
| 12.فکر کردن به زایمان، من را اذیت می‌کنم. |  |  |  |  |
| 13.من وقتی در مورد زایمان داستانی را می‌شنوم دچار نگرانی می‌شوم. |  |  |  |  |
| 14.من نگرانم که ناراحتی‌های جسمانی دوران بارداری، پس از زایمانم هم باقی بماند. |  |  |  |  |
| 15.من واقعاً می‌توانم احساساتم را با همسرم قسمت کنم. |  |  |  |  |
| 16.من در خصوص افزایش وزنم در دوران بارداری نگرانم. |  |  |  |  |

کلید محاسبه نمره مقیاس

موارد 3، 5 ، 6 ، 7، 9 ، 10 ، 11 ، 12 ، 13 ، 14 ، 16 باید اینگونه نمره دهی شوند ( 3=0 ، 2=1 ، 1=2 ، 0= 3)
